# Antibody responses to the host-protective *Taenia solium* oncosphere protein TSOL18 in pigs are directed against conformational epitopes

**DOI:** 10.1111/j.1365-3024.2009.01197.x

**Published:** 2010-06

**Authors:** E ASSANA, C G GAUCI, C T KYNGDON, A P ZOLI, P DORNY, S GEERTS, M W LIGHTOWLERS

**Affiliations:** 1 Department of Animal Health, Prince Leopold Institute of Tropical MedicineAntwerpen, Belgium; 2 Veterinary Clinical Centre, The University of MelbourneWerribee, Vic., Australia; 3 Faculty of Agronomy and Agricultural Sciences, University of DschangDschang, Cameroon

**Keywords:** conformational epitope, cysticercosis, pig, *Taenia solium*, TSOL18, vaccine

## Abstract

TSOL18 is a recombinant protein that has been shown in repeated experimental trials to be capable of protecting pigs against challenge infection with the cestode parasite *Taenia solium*. Antibodies raised by the vaccine are capable of killing the parasite in an *in vitro*culture and it is believed that antibody and complement-mediated killing of invading parasites is the major protective immune mechanism induced by vaccination with TSOL18. Investigations were undertaken to characterize whether the principal antibody specificities raised by TSOL18 in pigs were against linear or conformational determinants. TSOL18 was expressed in two truncated forms representing either the amino terminal portion or the carboxy terminal portion, with the two truncations overlapping in sequence by 25 amino acids. The original protein (designated TSOL18N^−^) and the two truncations (TSOL18N^−^-1 and TSOL18N^−^-2) were used in inhibition ELISA. TSOL18N^−^ was shown to be capable of completely inhibiting the binding of pig anti-TSOL18N^−^ antibodies to TSOL18N^−^ in ELISA. However, neither TSOL18N^−^-1 nor TSOL18N^−^-2, either alone or when combined together, was capable of inhibiting any detectable amount of reactivity of pig anti-TSOL18N^−^ antibodies with TSOL18N^−^. It is concluded that the dominant antibody specificities, and probably the host-protective specificities, of TSOL18 are conformational epitopes.

## Introduction

*Taenia solium* is a zoonotic parasite prevalent in many developing countries of Asia, Africa and the Americas (1–3). The parasite lifecycle involves pigs and humans. Transmission to pigs is through the ingestion of faeces from a human infected with the adult *T. solium* tapeworm, or items contaminated with such faeces. Control of the disease can be achieved by improvements in sanitary conditions concerning the disposal of human faeces and through prevention of pigs gaining access to human faeces. However, in many areas of the developing world where the disease is prevalent, these measures are unlikely to be implemented in the foreseeable future. Efforts have been made to control this disease, but few countries have been able to eradicate or reduce the infection level using approaches such as anthelmintic treatment of humans or pigs, restriction of roaming pigs, health education or meat inspection. Failure to control *T. solium* cysticercosis using these approaches in the last decades has indicated that eradication of this zoonosis will be difficult to achieve (4).

Vaccines have been proposed as a new approach to control pig cysticercosis and interrupt the life cycle of *T. solium* (5). Several candidate vaccines are now available (6–8). Antigens derived from the oncosphere lifecycle stage have been the most effective in inducing protection against experimental challenge infection with taeniid cestode parasites (9). Development of a vaccine against *Taenia ovis* infection in sheep (10) provided a model for identification of homologous antigens in related parasites (11). Subsequently, a number of effective vaccines have been developed based on oncosphere proteins expressed in *Escherichia coli* (9). Adoption of a similar approach for *T. solium* led to the discovery of the protein TSOL18, which has been found to induce between 99·3 and 100% protection in five experimental challenge trials carried out in four different countries (12,13, reviewed in Ref. 9). Investigations into the molecular aspects of gene structure and the translated protein sequences show that the various host-protective oncosphere antigens from different taeniid cestode species show common features in the structure of the proteins. These include a predicted secretory signal sequence and one or two copies of a fibronectin type III domain (FNIII; 13,14).

A principal host-protective immune mechanism induced by oncosphere antigens against taeniid cestode infections is antibody and complement-mediated killing of early stages in the development of the parasite in the intermediate host (9). Little is known about the nature of the host-protective epitopes associated with the various oncosphere proteins that are under development as practical vaccines. Knowledge of the nature of antigenic sites recognized by antibody is an important component in understanding the characteristics of a vaccine antigen and the development of associated immunological assays (15). Efforts to identify protective epitopes have, to date, not been successful (16–18). The antigen about which most information is available is the EG95 protein from the related parasite *Echinococcus granulosus*. Strong evidence has been obtained that the host-protective epitopes associated with this antigen are principally, or entirely, associated with the tertiary conformation of the protein and not associated with linear epitopes (17).

TSOL18 is undergoing further development in anticipation of its application in the control of transmission of *T. solium* through pigs. At present the vaccine comprises a purified recombinant protein. While this source of vaccine antigen may be effective, production of recombinant proteins is relatively expensive and an attractive alternative would be the use of a defined protective epitope produced synthetically. The use of such a precise epitope which corresponds to the specificity of a known protective antibody could induce the generation of antibodies similar to those elicited by the vaccine (19). There are many similarities between TSOL18 and the EG95 protein, including the presence of a secretory signal sequence followed by a single FNIII domain. In the experiments described in this study we use sera from pigs known to be protected against *T. solium* infection in *in vitro* assays with the TSOL18 protein and with truncated recombinant forms of TSOL18 to determine whether the host-protective anti-TSOL18 antibodies are associated with conformational determinants.

## Methods

### Preparation of TSOL18

The TSOL18 antigen used in these experiments was identical to the vaccine protein used in the successful vaccine trials described by Flisser *et al.* (7) and Gonzalez *et al.* (12) being an N-terminal truncation of the full length TSOL18 protein from which the 18 amino acid secretory signal sequence had been deleted. The nomenclature used here for this protein is TSOL18N^−^. The protein was expressed as a C-terminal fusion to glutathione *S*-transferase (TSOL18N^−^-GST; 12). TSOL18N^−^ was also expressed as a maltose binding protein (MBP) fusion by cloning and expressing TSOL18N^−^ cDNA into pMAL using standard procedures (20). Soluble TSOL18N^−^-GST or TSOL18N^−^-MBP were affinity purified from *E. coli* proteins using glutathione-sepharose (Amersham Bioscience, Uppsala, Sweden) or maltose beads (Biolabs, New England, UK) for the GST and MBP fusion proteins respectively. Control proteins were prepared from *E. coli* transformed with the pGEX or pMAL vectors according to the manufacturers’ instructions.

### Preparation of truncated TSOL18N^−^

**Figure 1:**
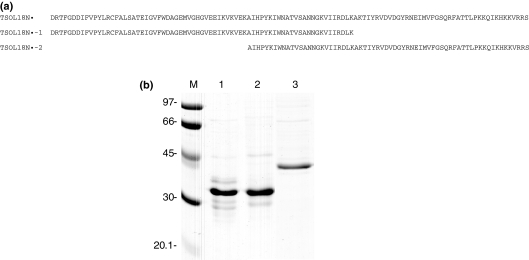
Amino acid sequence of *T. solium* TSOL18N^−^ and truncated proteins TSOL18N^−^-1 and TSOL18N^−^-2 (a) and the appearance of the proteins as GST fusions in SDS PAGE (b). M, molecular weight markers, 1 TSOL18N^−^-1, 2 TSOL18N^−^-2, 3 TSOL18N^−^.

Two truncated TSOL18N^−^ proteins (TSOL18N^−^-1, TSOL18N^−^-2) were expressed in *E. coli* as GST fusion proteins. PCR products were amplified from the TSOL18N^−^ cDNA template so as to express the truncated proteins shown in Figure 1a. Amplification of TSOL18N^−^ cDNA fragments was undertaken using the following primer pairs:

TSOL18-1, 5′GAA TTC GAC CGA ACA TTC GGC GAC G 3′, 5′CTC GAG TCA CTT CAA GTC TCT CTG ATG AC; TSOL18-2, 5′ GAA TTC GCA ATA CAC CCA TAC AAG ATC TG 3′, 5′ CTC GAG TCA GCA TTG CCT GCT CCG CGC 3′.

PCR was performed in a total volume of 50 μL containing 0·3 mm dNTP, 1 mm MgSO_4_, 1·25 Units Pfx DNA polymerase (Invitrogen, Carlsbad, CA, USA), 1 × Pfx reaction buffer (Invitrogen), primers 20 ng each and 0·1 ng pGEX-1TEX-TSOL18N^−^ template. Amplification was carried out in Perkin Elmer GeneAmp PCR Reactor 9600 (Perkin Elmer, Waltham, MA, USA) for 30 cycles consisting of 94°C for 30 s, 55°C for 30 s, 68°C for 1 min. Reaction products were ethanol precipitated, restriction digested with *Eco*RI and *XhoI,* separated using TAE-agarose gel electrophoresis and visualized using SYBR Green (Invitrogen). Stained bands were excised and purified using a Qiagen Minelute kit. *Eco*RI and *XhoI* digested TSOL18N^−^-1-DNA and TSOL18N^−^-2-DNA were ligated to previously *Eco*RI and *XhoI* digested pGEX-1TEX and transformed into *E. coli* BL21 DE3 using electroporation. DNA sequence of the clones was confirmed using dideoxy chain termination method using the Applied Biosystems ABI PRISM™ sequencing system and BIG DYE V3.1 terminator cycle sequencing kit. Sequencing results were analysed using DS Gene software (ACCELRYS, San Diego, CA, USA).

TSOL18N^−^-1 comprised the amino-terminal 71 amino acids of TSOL18N^−^ (Figure 1a) while TSOL18N^−^-2 consisted of the carboxy-terminal 66 amino acids of TSOL18N^−^ with the two truncated proteins overlapping each other by 25 amino acids. All the steps of production and purification of truncated antigens fused with glutathione *S*-transferase were identical to those used for the full length TSOL18N^−^-GST protein.

### Serum samples

Sera were obtained from two pigs immunized with TSOL18N^−^-GST (whole proteins) and two pigs immunized with the combined two fragments of TSOL18N^−^. The immunizations consisted of 200 μg of soluble antigens plus 1 mg of the adjuvant Quil-A (Superfos Biosector, Vedbaek, Denmark) given a month apart. Sera were collected at 2 weeks post second immunization. In addition, sera from two Cameroonian pigs collected 4 weeks after experimental infection with *T. solium* eggs (21), were obtained from the bank sera of the Animal Health Department of the Institute of Tropical Medicine, Belgium.

### Oncosphere antigen

#### Enzyme-linked immunosorbent assays

TSOL18N^−^-specific antibody titres were determined using enzyme-linked immunosorbent assay (ELISA) using TSOL18N^−^-MBP as antigen (to differentiate anti-TSOL18N^−^ antibodies from those raised against the GST component of TSOL18N^−^-GST). Optimal concentrations of antigen and enzyme conjugated secondary antibody were determined in checker board titrations. ELISA plates (Nunc®, Polysorb, Roskilde, Denmark) were coated overnight at 4°C with TSOL18N^−^-MBP (5 μg/mL) in carbonate buffer (0·06 m, pH 9·6, 100 μL/well). The plates were washed once with phosphate-buffered saline containing 0·05% Tween 20 (PBST) and blocked by incubation with 150 μL per well of 2% new born calf serum in phosphate-buffered saline-0·05% Tween 20 (NBCS-PBST) for 1 h at 37°C. Plates were emptied and 100 μL of test serum samples serially diluted in NBCS-PBST were added and incubated at 37°C for 1 h after which the plates were washed five times with PBST and incubated with 100 μL per well of rabbit anti-porcine IgG conjugated to horseradish peroxidise (HRP) (Sigma-Aldrich Corp, St Louis, MO USA) diluted at 1/20 000 in NBCS-PBST at 37°C for 1 h. Subsequently the plates were washed as above and chromogen/substrate solution (Ortophenylene diamine and H_2_O_2_) was added (100 μL/well) and incubated at 37°C for 15 min. The reaction was stopped by addition of 50 μL of 4N H_2_SO_4_ to each well. Optical densities (OD) were measured at 492 nm using a microplate reader (Multiscan EX; Thermo Scientific, Waltham, MA, USA). Titres were calculated as the dilution at which the sera had an OD of 1·0.

#### Inhibition ELISA

Inhibition ELISA were performed using two methodologies. Tests using serially diluted antigen and constant amount of antibodies were undertaken as follows. ELISA plates (Microlon 655061; Greiner Bio-One, Longwood, FL, USA) were coated and blocked in the manner described above. Antigens TSOL18N^−^-GST, TSOL18N^−^-MBP, TSOL18N^−^-1 or TSOL18N^−^-2 were serially diluted in PBST to obtain 11 concentrations from 3·21 pmol/mL to 3290·55 pmol/mL. In total 150 μL of antisera (1/100 in PBST-1% sodium caseinate-20%*E. coli* lysate) was added to 150 μL of each serial dilution of antigen and incubated at 37°C for 2 h. A total of 100 μL of each of the inhibition mixtures was added to empty wells of an ELISA plate that had previously been coated with antigen (as for standard ELISA) and subsequently incubated at 37°C for 1 h. Plates were washed five times with PBST, incubated with 100 μL per well of goat anti-porcine IgG conjugated to horseradish peroxidise (HRP) (Serotec, Oxford, UK), diluted at 1/5000 in 1% sodium caseinate-phosphate-buffered saline-0·05% Tween20 (SC-PBST) and incubated at 37°C for 1 h. The plates were washed again and TMB chromogen/substrate solution in phosphate/citrate buffer (0·2 m dibasic sodium phosphate, 0·1 m citric acid, pH 5; 1 mm 3,3′5,5′ tetramethylbenzidine; 3·6 × 10^−2^% of H_2_O_2_) was added (100 μL/well) and incubated at 37°C for 25 min. The reaction was stopped by adding 50 μL of 2 m H_2_SO_4_ to each well. Optical densities (OD) were measured at 450 nm using a microplate reader (Dynex Technologie Ltd, Worthing, UK).

Inhibition ELISA using a constant amount of antigen and serially diluted antiserum were undertaken using similar procedures to those described above but a constant amount (10 μg) of the TSOL18 antigens was added to a serial dilution of antiserum.

## Results

TSOL18N^−^-1-GST and TSOL18N^−^-2-GST were expressed in *E. coli* as soluble fusion proteins that were successfully purified on glutathione agarose and migrated according to their predicted sizes in SDS PAGE (Figure 1b).

**Figure 2:**
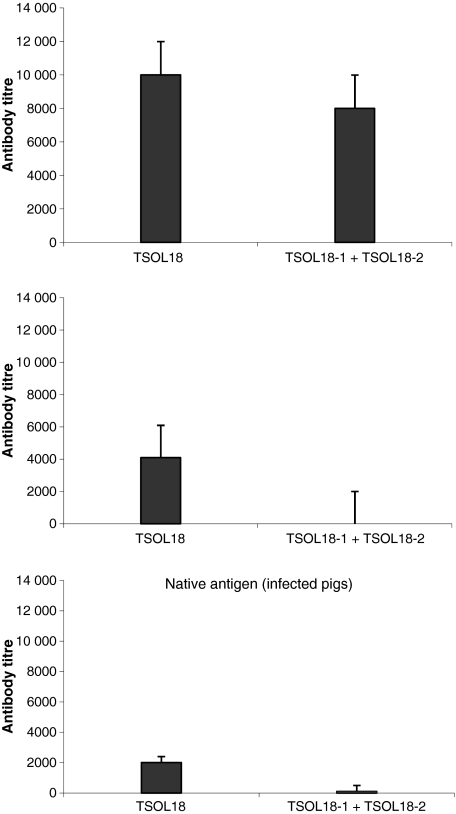
Antibody titres in pigs 2 weeks after a second immunization with either TSOL18N^−^-GST, a combination of TSOL18N^−^-1-GST plus TSOL18N^−^-2-GST or 4 weeks post infection with *T. solium* eggs. Panel headings indicate the antigen used to raise antisera while the *y*-axis legends indicate the antigens used in ELISA. Top panel shows reactivity in ELISA (±standard deviation) with the homologous antigens (TSOL18N^−^-GST, TSOL18N^−^-1-GST and TSOL18N^−^-2-GST). Centre panel: specific reactivity to the TSOL18N^−^-encoded portions of the proteins measured using the associated MBP fusion proteins as antigen, or oncospheres, in ELISA respectively. Bottom panel: reactivity of *T. solium* infected pigs (exposed to the TSOL18 native protein) reacted against TSOL18N^−^ MBP fusion proteins in ELISA.

Antisera raised against TSOL18N^−^-GST, TSOL18N^−^-1-GST and TSOL18N^−^-2-GST were found to have detectable levels of specific antibody in ELISA (Figure 2). The level of reactivity measured in ELISA using TSOL18N^−^-MBP as antigen was greater in animals immunized with TSOL18N^−^ than it was with sera from animals vaccinated with the two truncated proteins (Figure 2).

**Figure 3:**
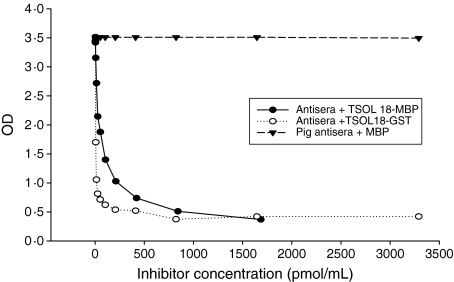
Inhibition ELISA where serial dilutions of TSOL18N^−^-GST, TSOL18N^−^-MBP or MBP proteins were used to inhibit the binding of pig anti-TSOL18N^−^-GST antisera reacting against TSOL18N^−^-MBP as antigen. Serial dilutions of inhibitory proteins were from 3·21 pmol/mL to 3290·55 pmol/mL. Both TSOL18N^−^-GST, TSOL18N^−^-MBP were effective inhibitors in the assay whereas the control protein (MBP) caused no inhibition.

**Figure 4:**
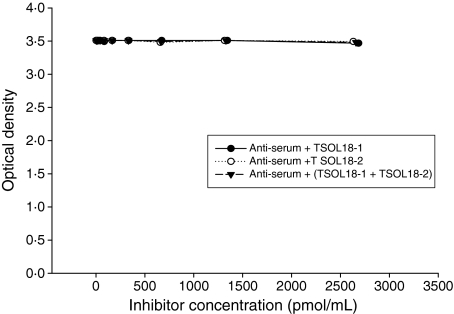
Inhibition ELISA where serial dilutions of TSOL18N^−^-1-GST and TSOL18N^−^-2-GST were incubated with pig anti-TSOL18N^−^-GST antisera prior to undertaking ELISA against TSOL18N^−^-MBP as antigen. The TSOL18N^−^-1-GST and TSOL18N^−^-2-GST proteins were used either individually or combined together. Neither truncated form of the TSOL18 protein was able to inhibit any detectable amount of reactivity between pig anti-TSOL18N^−^-GST and TSOL18N^−^.

**Figure 5:**
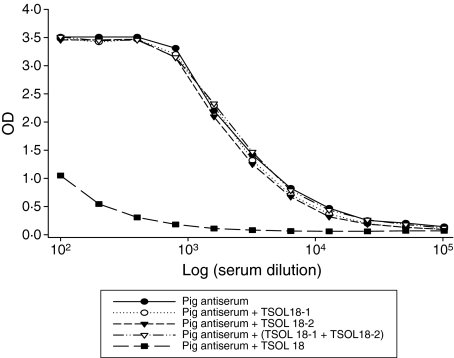
Inhibition ELISA where the inhibiting protein was kept at a constant concentration and exposed to serially diluted antisera prior to reaction in ELISA with TSOL18N^−^-MBP as antigen. TSOL18N^−^-GST was an effective inhibitor of anti-TSOL18N^−^-1-GST antibody reactivity whereas neither TSOL18N^−^-1-GST nor TSOL18N^−^-2-GST either singly or together were able to induce any detectable level of inhibition.

Both TSOL18N^−^-GST and TSOL18N^−^-MBP were potent inhibitors of pig specific antibody reactivity in ELISA (Figures 3 and 5) irrespective of whether the assays were performed using a constant concentration of antisera and a varying amount of inhibitor protein (Figure 3) or where differing amounts of anti-TSOL18N^−^-GST specific antibody was exposed to 10 μg of inhibitor protein (Figure 5). However, the proteins TSOL18N^−^-1-GST and TSOL18N^−^-2-GST were unable to inhibit any detectable level of reactivity of anti-TSOL18N^−^ antibodies binding to the TSOL18N^−^ protein (Figures 4 and 5).

## Discussion

The TSOL18N^−^ protein was an effective inhibitor of anti-TSOL18N^−^ antibody reactivity in inhibition ELISA. TSOL18N^−^ completely inhibited reactivity at concentrations above approximately 1000 pmol/mL of inhibitor antigen under the conditions of ELISA used in this study (Figure 3). In contrast, the TSOL18 truncations TSOL18N^−^-1 and TSOL18N^−^-2 did not inhibit reactivity between anti-TSOL18N^−^ antibody and the homologous antigen.

The amino acid sequences of TSOL18N^−^-1-GST and TSOL18N^−^-2 overlapped in the central region of the TSOL18N^−^ protein by 25 amino acids (Figure 1). TSOL18N^−^-1 comprised the 71 amino acid amino-terminal portion of TSOL18N^−^ while TSOL18N^−^-2 comprised the 66 amino acid carboxy-terminal portion of TSOL18N^−^. Antisera raised in pigs to TSOL18N^−^ are known to have some level of antibodies specific for linear determinants of the TSOL18N^−^ protein (18). It could be expected that these linear epitopes of TSOL18N^−^ would be present on the TSOL18N^−^-1 and TSOL18N^−^-2 proteins. The data presented in this study would suggest that the great majority of the specific antibodies induced in pigs following immunization with TSOL18N^−^ are directed against conformational determinants rather than linear determinants, and that these conformational determinants are not presented by fragments of TSOL18N^−^ representing either the amino two-thirds of the protein or the carboxy two-thirds.

Similar data were obtained by Woollard *et al.* (17) when examining the nature of the antigenic epitopes of the protective oncosphere antigen EG95 of *E. granulosus*. In this case, three overlapping proteins were examined, representing the amino terminal, central and carboxy terminal portions of EG95. None of these proteins, either alone or as a combination, was able to inhibit binding to EG95 in ELISA of the host-protective antibodies in the sera of sheep vaccinated against EG95. Vaccination of sheep with the three overlapping proteins, representing the entire EG95 amino acid sequence, failed to induce protection against a challenge infection with *E. granulosus* whereas vaccination with the complete EG95 polypeptide induced near total protection (17). The conclusion drawn for EG95 was that most of the host-protective antibodies raised following EG95 vaccination were against conformational rather than linear determinants. EG95 and TSOL18 are both host-protective oncosphere antigens of taeniid cestode parasites, both contain a single FNIII domain and both appear to have the host-protective epitopes associated with conformational epitopes.

Infection with taeniid cestodes induces concomitant immunity, that is immunity against re-infection with the same species of parasite while those parasites establishing during the initial infection are unaffected (22). Much evidence exists to indicate that a major protective mechanism, if not the only protective mechanism, induced by an initial infection or using vaccination with oncosphere antigens is antibody and complement-mediated attack on the parasite during or immediately after the oncosphere invades the host tissues (reviewed in Ref. 9,22). Specific antibodies in the sera of pigs vaccinated with TSOL18N^−^ (23) or sheep vaccinated with EG95 (17) are able to kill *T. solium* or *E. granulosus* oncospheres respectively, in an *in vitro* culture. There are no published data concerning the colostral transfer of immunity induced by recombinant oncosphere antigens, however, either vaccination with native oncosphere antigens or an initial infection with a taeniid cestode parasite induces antibodies that are capable of transferring complete protection against infection in naïve recipients (reviewed in Ref. 9,22). The data concerning the nature of the major antibody specificities induced in pigs by TSOL18N^−^ or in sheep induced by EG95 suggests that the major protective epitopes of the proteins are conformational.

Options for the development of a synthetic peptide based vaccine are limited where the protective epitope(s) are conformational. Phage display mimotope technology has been applied to the characterisation of epitopes of conformational epitopes of EG95 (24,25). One mimotope was identified which was capable of affinity purifying protective antibody specificities, however, this represented a minor component of the protective antibody produced by the EG95 vaccine (25). An alternative technology that may be applied to the further characterization of the antigenic epitopes on TSOL18N^−^ is the use of monoclonal antibodies. Preliminary investigations (E. Assana, unpublished data) have shown that mice raise antibodies against TSOL18N^−^ have similar characteristics to pig antibodies against TSOL18N^−^. TSOL18N^−^ protein was able to fully inhibit the binding of mouse anti-TSOL18N^−^ antibodies whereas TSOL18N^−^-1 and TSOL18N^−^-2 failed to inhibit any reactivity of the mouse antisera against TSOL18N^−^ and the homologous antigen in ELISA. These findings suggest that the major antibody specificities raised in mice against TSOL18N^−^ may be similar to those raised in vaccinated pigs and hence monoclonal antibody technology may provide a suitable method to further characterize the conformational epitopes of TSOL18N^−^.
